# Health coaching by telephony to support self-care in chronic diseases: clinical outcomes from The TERVA randomized controlled trial

**DOI:** 10.1186/1472-6963-12-147

**Published:** 2012-06-10

**Authors:** Kristiina Patja, Pilvikki Absetz, Anssi Auvinen, Kari Tokola, Janne Kytö, Erja Oksman, Risto Kuronen, Timo Ovaska, Kari Harno, Mikko Nenonen, Tom Wiklund, Raimo Kettunen, Martti Talja

**Affiliations:** 1 National Institute of Health and Welfare, Helsinki, Finland; 2 School of Health Sciences, University of Tampere, Tampere, Finland; 3 Pfizer Oy, Helsinki, Finland; 4 Joint Authority for Päijät-Häme Social and Health Care, Lahti, Finland; 5 Department of Social and Health Management, University of Eastern Finland (Kuopio Campus), Kuopio, Finland; 6 Hartola Health Care Center Hartola, Finland and and Finnish Medical Association, Helsinki, Finland

## Abstract

**Background:**

The aim was to evaluate the effect of a 12-month individualized health coaching intervention by telephony on clinical outcomes.

**Methods:**

An open-label cluster-randomized parallel groups trial. Pre- and post-intervention anthropometric and blood pressure measurements by trained nurses, laboratory measures from electronic medical records (EMR). A total of 2594 patients filling inclusion criteria (age 45 years or older, with type 2 diabetes, coronary artery disease or congestive heart failure, and unmet treatment goals) were identified from EMRs, and 1535 patients (59%) gave consent and were randomized into intervention or control arm. Final analysis included 1221 (80%) participants with data on primary end-points both at entry and at end. Primary outcomes were systolic and diastolic blood pressure, serum total and LDL cholesterol concentration, waist circumference for all patients, glycated hemoglobin (HbA_1c_) for diabetics and NYHA class in patients with congestive heart failure. The target effect was defined as a 10-percentage point increase in the proportion of patients reaching the treatment goal in the intervention arm.

**Results:**

The proportion of patients with diastolic blood pressure initially above the target level decreasing to 85 mmHg or lower was 48% in the intervention arm and 37% in the control arm (difference 10.8%, 95% confidence interval 1.5–19.7%). No significant differences emerged between the arms in the other primary end-points. However, the target levels of systolic blood pressure and waist circumference were reached non-significantly more frequently in the intervention arm.

**Conclusions:**

Individualized health coaching by telephony, as implemented in the trial was unable to achieve majority of the disease management clinical measures. To provide substantial benefits, interventions may need to be more intensive, target specific sub-groups, and/or to be fully integrated into local health care.

**Trial registration:**

ClinicalTrials.gov Identifier: NCT00552903

## Background

Diabetes and cardiovascular diseases represent large and costly chronic healthcare challenges [1]. Preventative measures can effectively reduce costs [2]. Despite differences between different conditions, the expectations on the patients are similar: to cope with multiple medications and co-morbidities, to alter behavior, to deal with social and psychological impacts of symptoms and to interact with medical care [3,4].

Health care providers have a difficult task in trying to manage chronic disease care in complex service systems that are poorly designed to motivate, equip and empower patients to behavior changes [5-7]. Resources should aim at maximized health gains, and this requires reorientation of services [8]. High expectations are put on information technology solutions that have been shown highly effective in promoting lifestyle changes [9]. So far, comprehensive efforts to assess the impact of incorporating a range of IT tools in chronic disease management have been targeting single disease groups, such as CHD [10,11], heart failure [12] or diabetes [13,14] Taylor et al. 2003, but studies with several disease groups and/or co-morbidities are lacking.

While technology can be an effective way to improve reach of disease management interventions, still the content is more important. Health coaching, a collaborative process characterized by motivational communication, patient-defined goals related to disease management, and patient acceptance of accountability for decisions made [15] can utilize different sets of self-management tools (SMTs) to promote adoption of an active role in self-care by the patient [16]. Health coaching can improve quality, effectiveness and cost-effectiveness of disease management [17]. The TERVA trial is the first large randomized controlled trial to simultaneously evaluate tele-coaching in a real-world health care setting in three patient groups: congestive heart failure (CHF), coronary artery disease (CAD) and type 2 diabetes mellitus (T2D). The aim of the trial was to assess the effect of health coaching on clinical outcomes (risk determinants) after one-year intervention.

## Methods

### Trial design

The TERVA study is a randomized, open-label, parallel groups trial comparing health coaching and usual care. The primary end points were defined as 10-percentage point difference between arms in increase in the proportion of participants reaching the target level in five global and two patient-group specific clinical parameters at 12 months (Table 1). The targets were set in accordance with Finnish evidence-based guidelines.

**Table 1:** Primary and secondary end points of TERVA trial

**Primary end points**
▪ Provider-measured BP ≤140/85 mmHg
▪ Total cholesterol ≤4.5 mmol/L
▪ LDL ≤2.5 mmol/L
▪ Waist circumference ≤94 cm for men and ≤80 cm for women – later revised as 90 cm for women and 100 cm for men based on national guidelines
For congestive heart failure an additional end-point:
▪ Improved or maintained NYHA class
For participants with T2D:
▪ HbA_1c_ ≤7%

### Measures

Research nurses, unaware of the allocation, measured blood pressure and waist circumference in both arms. The laboratory results were extracted from the electronic medical records (EMR) at both entry and end of the intervention (at entry between 3 months before to 1 month after and at end 11 to 15 months from date of consent). NYHA-class was obtained from study questionnaires at entry and end of follow-up.

### Identification and enrollment

Patients were enrolled from Päijät-Häme in the Southern Finland, a region with a population of 212,000. The target population was initially identified from primary care and hospital registries and records, followed by a detailed assessment of medical records (Table 2). Patients with more than one condition were enrolled in the following hierarchy: CHF - CAD - T2D, so CHF patients could have CAD and/or T2D, but not the other way around. All eligible patients were sent an information letter and a consent form in four batches during a 12-month period in 2007–2008 with one reminder for non-responders followed by a telephone call. Of the 2594 eligible patients 59.2% (1535) gave consent and were invited for an examination and interview by the research nurse, and 1225 (79.8%) completed it. The final analysis included 1221 patients (80%) having data on primary end-points both at entry and at end of follow-up. 1215 had both baseline and end of study measurements of waist circumference and blood pressure available (812 or 87% of committed patients in the intervention arm and 403 or 87% in the control arm). Laboratory measures of lipids at both time points were available in EMRs only for a fifth of the patients, and HbA_1c_ for 54% of the patients with diabetes. The age and sex distribution of the drop-outs did not differ from the analyzed patients (mean ages 65.0 vs. 64.8 years, 60.6% vs. 58.1% men). There were no substantial differences between participants and drop-outs in the primary end-points at baseline.

**Table 2:** Eligibility and exclusion criteria of TERVA trial

**Eligibility criteria for enrollment included:**
1. Residents in the region of Päijät-Häme aged 45 years or older
2. One of the following diagnose
a. Heart failure with NYHA II or III, and a history of hospital admission for heart failure within the last 2 years
b. History of myocardial infarction or cardiac revascularization procedure, and one of the following (treated or untreated): blood pressure above 140/85 mmHg, total serum cholesterol concentration
>4.5 mmol/L, serum LDL concentration >2.5 mmol/L
c. T2D on medication and serum HbA_1c_ >7% without clinically evident cardiovascular diseases e.g. MI, stroke, peripheral vascular disease
Exclusion criteria:
● Inability to cooperate or participate
● Pregnancy
● Life expectancy less than 1 year
● Patients with major elective surgery planned within 6 months
● Patient has had major surgery within the last 2 months

### Randomization

**Figure 1:**
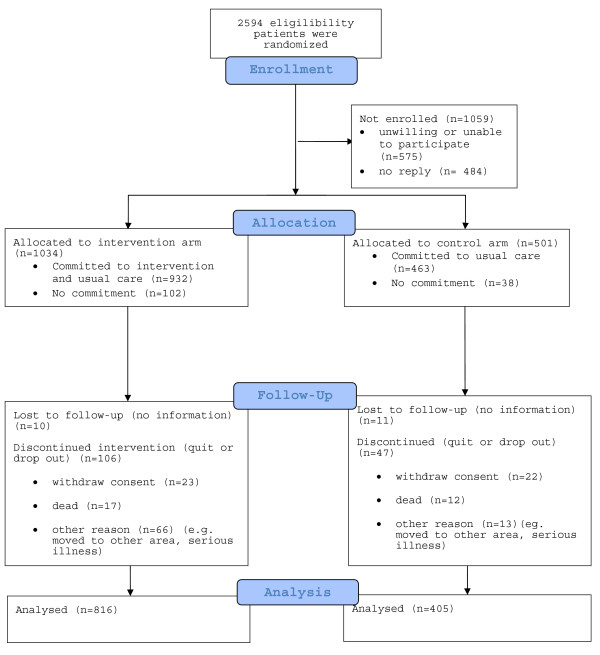
**Flow Diagram.** Distribution of study population from those filling inclusion criteria by the healthcare charts to those completed the intervention.

A cluster design was used to accommodate the effects of individual health coaches with multiple patients. The randomization algorithm was based on computer-generated random numbers. A stratified randomization with permuted blocks was used to ensure balanced distribution within disease group and municipality between the arms. A Zelen type randomization (2:1 ratio for intervention/control arm) was performed prior to consent (Figure 1).

### Intervention

**Figure 2:**
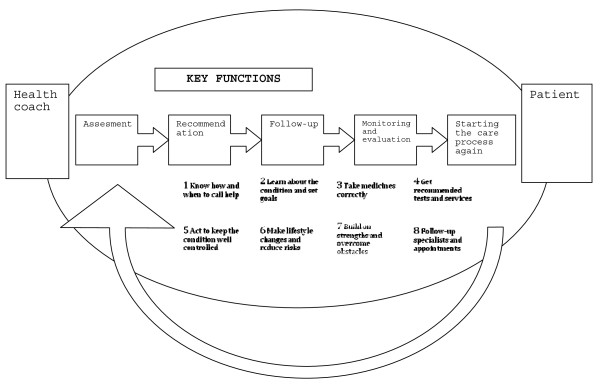
Pfizer Health Solutions has developed a tele-coaching intervention with 5 key functions and 8 recommendations to engage, inform, involve, and empower the patients in self-care.

Health coaching was delivered from November 2007 by seven experienced certified nurses or public health nurses. They were trained for four weeks in a tele-coaching model initially developed by Pfizer Health Solutions (PHS) but modified for the Finnish health care system. Patients in the intervention group were called monthly, altogether 10–11 times. After a brief engagement call, there was one broader needs assessment call, followed by monthly coaching calls and finally an evaluation call. In between the coaching calls there was an opportunity for brief follow-up calls, but these were rarely used. The coaching call topics were based on 8 key recommendations of the program, with variations due to individual patient’s preferences (Figure 2). The behavior change component integrated behavior change techniques from the Self-Regulation Theory and supported by evidence, i.e., self-monitoring, goal setting, action planning, and feedback [18]. After the first two months, quality assurance measures were taken in the form of listening to randomly selected 2–3 calls from each coach. Call length was also monitored. Calls were found to be long, typically up to 60 min, and they were based on a coach driven information provision model, and very little concrete goal setting and action planning was done. To improve quality, an explicit structure following the self-regulation model was developed jointly with the coaches, and the maximum number of topics to be tackled during one call, was limited to three. Also, coaches were further trained in Motivational Interviewing techniques of active listening, and using open questions, reflection and summaries [19], and they all received two individual supervision sessions in self-monitoring and developing their coaching practices. With these measures, quality (defined as use of structure and Motivational Interviewing techniques, and concrete actions as outcomes of the calls) was improved while call length decreased to approximately 30 min. Self-care books prepared in collaboration with the Finnish Heart Association and the Finnish Diabetes Associations supported the coaching, and the coach had access to the patients’ EMRs. Both trial arms continued to receive routine care.

### Statistical methods

A sample size of 1250 patients was calculated to provide adequate statistical power (1-β ≥ 0.8) for detecting a 10 percentage point difference between the intervention arms (with 6 coaches) with conservative assumptions (α = 0.05 two-sided, 50% of the patients in the control arm would reach target, a 10% drop out rate and 10% of the subjects not evaluable at the end of the trial), as long as the intracluster correlation did not exceed 0.01 [20].

Data analyses were conducted using multilevel methods (generalized linear mixed models) to account for the clustered design. The trial data had a two-level structure, where the health coaches constituted an upper level, within which the individual patients were distributed allowing for correlation at individual level within a cluster (variance components at the two levels).

A modified intention to treat analyses by trial arm was employed including all patients with data at entry and at the end of the 12-month follow-up. No substantial imbalance at baseline was found in the primary end-point variables between the arms (Table 3).

**Table 3:** Baseline data available from patients who were allocated to the study (intervention = 1034, control = 501)

	**Type 2 Diabetes**		**Coronary artery disease**		**Congestive heart failure**	
	**Intervention**	**Control**	**Intervention**	**Control**	**Intervention**	**Control**
Number of patients	770	359	172	97	92	45
Age (years)	64.6 (9.4)	65.6 (9.5)	65.4 (9.4)	66.0 (8.6)	67.3 (7.9)	62.4 (7.7)
Sex (% male)	58.3	54.0	69.2	73.2	65.2	64.4
Age at self reported year of diagnosis	54.3 (11.1)	56.1 (11.8)	60.8 (10.2)	61.3 (10.1)	63.9 (7.9)	56.8 (9.3)
	311	145	121	69	51	25
Self-reported duration of disease	10.3 (8.4)	9.2 (8.2)	4.7 (6.6)	4.8 (7.6)	4.4 (5.4)	4.2 (3.7)
	302	142	120	68	51	25
Body mass index	32.3 (6.2)	31.9 (5.7)	28.3 (4.1)	28.8 (5.1)	30.2 (6.6)	29.9 (6.9)
	727	338	164	91	81	42
Waist circumference (M/F)	109.9 (14.4)/ 106.8 (15.5)	110.1 (12.8)/ 104.8 (15.1)	100.3 (11.0)/ 95.1 (12.8)	102.9 (11.7)/ 91.5 (12.6)	106.7 (12.8)/ 94.1 (14.6)	108.0 (17.9)/ 89.6 (17.3)
	429/298	182/156	116/47	66/25	55/25	25/16
Systolic blood pressure (mmHg)	143.4 (20.0)	143.1 (20.2)	137.6 (20.5)	139.9 (18.2)	132.9 (23.7)	127.6 (22.7)
	727	337	163	91	81	42
Diastolic blood pressure (mmHg)	84.2 (11.1)	84.1 (10.8)	81.0 (12.2)	82.1 (10.5)	81.3 (14.1)	80.2 (11.5)
	727	338	163	91	81	42
Serum total cholesterol (mmol/l)	4.4 (1.1)	4.4 (0.9)	3.8 (0.9)	3.8 (1.0)	4.0 (1.2)	4.0 (1.4)
	225	121	66	46	23	12
Serum HDL cholesterol (mmol/l)	1.2 (0.4)	1.3 (0.4)	1.4 (0.4)	1.3 (0.4)	1.1 (0.4)	1.3 (0.5)
	219	119	65	45	23	11
Serum LDL cholesterol (mmol/l)	2.3 (0.8)	2.4 (0.8)	1.9 (0.6)	1.9 (0.7)	2.1 (0.9)	2.2 (0.9)
	210	115	65	45	21	11
Lipid lowering medication (%)	24.7	20.3	60.5	55.7	38.0	22.2
	190	73	104	54	35	10
Daily smokers (%)	12.7	11.0	11.9	10.1	12.7	12.2
	88/691	36/327	19/160	9/89	10/79	5/41
Hb1Ac (%)	7.5 (1.1)	7.7 (1.7)				
	415	224				
Oral antidiabetic drug and insulin (%)	12.3	13.1				
	95	47				
Oral antidiabetic drug (%)	34.0	29.8				
	262	107				
Insulin (%)	16.8	16.7				
	129	60				
SCORE^1^	8.0 (6.3)	7.6 (7.9)				
	215	115				
NYHA^2^					2.4 (1.4)	2.5 (1.4)
					52	30

^1^ Score index includes: Sex, Age, Total Chol, HDL and BP, smoking status.

^2^ New York Heart Association index on angina pectoris.

### Ethical approval and trial number

Written informed consent was obtained from all participants prior to enrollment into the project. The study protocol was approved by the Ethics Committee of the PHSSHD and registered (ClinicalTrials.gov Identifier: NCT00552903).

## Results

In the intervention arm, 48.1% of the patients (156/324) initially above the target level of diastolic blood pressure of 85 mmHg reached this value, while for the control arm the proportion was 37.3% (62/166). The 10.8% (95% confidence interval (CI) 1.5–19.7%) difference in proportion of patients who reached the goal was statistically significant and gave a number needed to treat of 10 (CI 5–66). Of the patients with a systolic blood pressure above the target level of 140 mmHg at baseline, 35.9% (143/398) in the intervention arm and 31.0% (58/187) in the control arm reached the target (*p* = 0.24).

For waist circumference, the target was below 100 cm for men and 90 cm for women. The difference was not statistically significant (*p* = 0.08 combined, 0.07 for males and 0.65 for females) (Table 4). For patients with T2D, the goal for HbA_1c_ there was no difference between intervention and control group (Table 4).

**Table 4:** Proportion (%) of those patients reaching targets in primary end points among those exceeding these values at baseline in the analysed population (intervention = 816, controls = 405)

	**Type 2 Diabetes**		**Coronary artery disease**		**Congestive heart failure**	
	**Intervention**	**Control**	**Intervention**	**Control**	**Intervention**	**Control**
Hb1Ac (<7%)	30.2% (n=65/215)	29.7% (n=27/91)				
Waist circumference (<90cm women, <100cm men)	9.8% (n=46/470)	5.1% (n=12/234)	11.1% (n=8/72)	9.5% (n=4/42)	10.8% (n=4/37)	15.8% (n=3/19)
Systolic blood pressure (<140mmHg)	32.7% (n=107/327)	35.8% (n=53/148)	47.1% (n=24/51)	16.1% (n=5/31)	60.0% (n=12/20)	0% (n=0/8)
Diastolic blood pressure (<85mmHg)	45.5% (n=120/264)	37.7% (n=49/130)	56.4% (n=22/39)	26.1% (n=6/23)	66.7% (n=14/21)	53.8% (n=7/13)
Serum total cholesterol (<4.5mmol/l)	30.7% (n=23/75)	35.0% (n=7/20)	77.8% (n=7/9)	100% (n=3/3)	100% (n=1/1)	0% (n=0/1)
Serum LDL cholesterol (<2.5mmol/l)	43.4% (n=29/67)	47.4% (n=9/19)	75.0% (n=6/8)	100% (n=2/2)	100% (n=1/1)	(n=0)
NYHA class (similar or improved)					83.9% (n=26/31)	93.3% (n=14/15)
Target reached in at least one primary endpoint*	47.8% (n=276/578)	44.8% (n=125/279)	49.5% (n=55/111)	43.8% (n=28/64)	75.5% (n=40/53)	65.4% (n=17/26)

*Intervention: 50.0% (371/742), Control: 46.1% (170/369), *p* = 0.217.

The goal for total cholesterol reduction was reached more often in control arm than in intervention arm (*p* = 0.64) as was the LDL cholesterol target (≤2.5 mmol/l) (*p* = 0.68). For patients with CHF, NYHA class remained similar or improved in both arms (*p* = 0.39). The proportion of patients achieving at least one of the defined primary objectives was 50.0% (371/742) in the intervention and 46.1% in the control arm (170/369, *p* = 0.22). Within the intervention arm, no substantial differences were found between subjects assigned to different nurses (intracluster correlation 0.01).

## Discussion

The TERVA trial was carried out in a real life setting and aimed at increasing the proportion of intervention patients reaching at least one of the predefined targets (blood pressure, HbA_1c_, waist circumference, NYHA class or total cholesterol) by 10% compared to controls. There was a **small**, non-significant improvement in the proportion of patients who reached at least one of the primary endpoints for both the whole study population, and for each of the disease area subgroups separately. However, the difference reached the predefined 10% difference between the groups only for the CHF patients. An encouraging finding is the high adherence, nearly 90% of the patients remained in the trial during the intervention (similar to the control arm). Further analysis of the intervention arm will define how well patients could achieve the goals that they actually set at the beginning of the intervention.

Chronic disease management is a complex process urging multiple simultaneous changes in self-care, in health behavior, and in the interaction with medical care [3,21]. A complex intervention such as ours that targets these multiple behaviors cannot be compared to single-behavior interventions such as smoking cessation, medication adherence, or physical activity interventions. Despite these methodological complexities, little differences were found between subjects assigned to different nurses, indicating consistency in delivering the intervention. Further, health behavior changes may have a delayed impact or may impact the risk of cardiovascular diseases independently of clinical outcomes [19]. These reasons may partly explain that we did not meet our study objectives. Another possibility is that the intensity of the intervention was too low to sufficiently cover multiple behaviors, as recent evidence suggests that telephony interventions targeting only physical activity or/and diet produce most favorable effects when the number of calls is 12 or more [9]. Several previous studies have assessed the effect of telephony interventions on similar outcomes as ours [6,7,14,22]. Also these trials have shown modest improvement in clinical and health behavior outcomes.

This study aimed to evaluate an intervention within the public health care system and occupationally active patients were underrepresented, as they are mostly covered by occupational health services [23], and retired patients with more severe disease are overrepresented. The T2D patients in the trial (selected based on HbA_1c_ >7% within 6 months prior to inclusion) represented approximately one third of the T2D patients in the region [24,25]. Of them 28% had HbA_1c_ >7% at the start of the intervention, which is comparable to the population-based studies of T2D patients [24], suggesting that the participants are representative of the target population. Davidson concluded in his review the key success factor in diabetes care being specially trained nurses or pharmacists and perhaps one reason for modest results was that those in treatment were receiving already specialist nurse care [4] and added value of telephony was limited.

We included three different disease areas with variable disease severity. The mean HbA_1c_ was only 7.5% in intervention arm and 7.7% in control arm, with 28% and 25% with baseline HbA_1c_ >7 respectively, and disease history of 9.2 and 10.3 years. The large proportion of T2D patients with HbA_1c_ at the target level at enrollment was due to the fact that the patients were originally screened from primary care EMRs, and had frequently improved by the time of enrollment, which could be up to 6 months later. Also, the end of study HbA_1c_ measurement could potentially be up to 10 months after the intervention. The abstraction of the laboratory data from EMRs instead of a strict measurement protocol was motivated by the pragmatic nature of the trial, but in the low proportion of subjects with such data at the end of the study reduced the power (despite reaching the target sample size) and could introduce bias, as assessments were not prescribed randomly. This limitation renders the findings related to laboratory data difficult interpret meaningfully. Further, the targets for primary end-points, for instance waist circumference, which were based on systematic reviews of behavioral risk factor and disease management interventions, may have been too stringent [26]. Finally, the intervention was not coordinated with other health care providers, but rather added on top of the existing services. Some specialist diabetes nurses expressed a concern that health coaching was challenging their professional role, but no assessments were carried out to objectively measure health professionals’ perceptions of the coaching program. Therefore, we can only speculate on the effect of the perceived competition on the results. However, it should be emphasized that the changes that were detected under these circumstances, demonstrate effects achieved in a real life setting.

## Conclusions

The results of this trial are inconclusive, as we did meet the primary end-point for diastolic blood pressure only with non-significant improvement in systolic blood pressure and waist circumference and no improvement in glycemic control, cholesterol or NYHA class. The overall lack of efficacy of health coaching may be related to the target population, coaching procedures and the duration of the follow-up time, and will be further explored in longer follow-up and sub-group analyses, as well as analysis of behavioral outcomes. Methodological factors and too strict primary targets may contribute to inability to meet all the predetermined primary objectives. Further, the primary analysis focused on efficacy, and analysis on resource utilization and cost-efficacy need to be performed to fully clarify the role of health coaching by telephone in this setting.

## Competing interest

P. Absetz trained health coaches (paid by Pfizer Oy). A. Auvinen’s and K. Tokola’s institution received grants from Joint Authority for Päijät-Häme Social and Health Care, Sitra, TEKES, and Pfizer Oy. J. Kytö was Medical Advisor for Pfizer Oy. Tom Wiklund was Medical Director of Pfizer Oy and had stock options of Pfizer Inc. Timo Ovaska was employee of Pfizer Oy. M. Talja’s institution received support for travel to meetings and fees for participation in review activities, and outside the submitted work for board membership, consultancy, employment, expert testimony and grants.

## Authors’ contributions

MT, AA, RKe, KP, RKu and TO participated in the design of the study (including conception, aims and procedures). EO, PA and TO took part in implementation of intervention. TW, KP, KH, MT, MN, RKe, RKu, PA and AA had oversight of the intervention throughout the study and the analysis, and planned the analysis. KT carried out data management and statistical analysis. All authors contributed to presentation and interpretation of findings. KP, AA and JK wrote the first draft. AA, KH, KP, JK, MN, MT, RKu, TW and PA took part in revision of the text for important intellectual content. All authors have approved the manuscript.

## Pre-publication history

The pre-publication history for this paper can be accessed here:

http://www.biomedcentral.com/1472-6963/12/147/prepub
